# Virtual reality as a teaching method for resuscitation training in undergraduate first year medical students during COVID-19 pandemic: a randomised controlled trial

**DOI:** 10.1186/s12909-022-03533-1

**Published:** 2022-06-22

**Authors:** Parisa Moll-Khosrawi, Alexander Falb, Hans Pinnschmidt, Christian Zöllner, Malte Issleib

**Affiliations:** 1 Department of Anaesthesiology, University Medical Centre Hamburg-Eppendorf, Martinistr. 52, 20246 Hamburg, Germany; 2 Center for Experimental Medicine, Institute of Medical Biometry and Epidemiology, University Medical Centre Hamburg-Eppendorf, Martinistr. 52, 20246 Hamburg, Germany

**Keywords:** Virtual reality, Basic Life Support, Chain of survival, Improvement of procedural skills, Learning gain

## Abstract

**Background:**

Virtual reality (VR) is a computer-generated simulation technique which yields plenty of benefits and its application in medical education is growing. This study explored the effectiveness of a VR Basic Life Support (BLS) training compared to a web-based training during the COVID-19 pandemic, in which face-to-face trainings were disrupted or reduced.

**Methods:**

This randomised, double-blinded, controlled study, enrolled 1^st^ year medical students. The control group took part in web-based BLS training, the intervention group received an additional individual VR BLS training. The primary endpoint was the no-flow time-an indicator for the quality of BLS-, assessed during a structural clinical examination, in which also the overall quality of BLS (secondary outcome) was rated. The tertiary outcome was the learning gain of the undergraduates, assessed with a comparative self-assessment (CSA).

**Results:**

Data from 88 undergraduates (*n* = 46 intervention- and *n* = 42 control group) were analysed. The intervention group had a significant lower no-flow time (*p* = .009) with a difference between the two groups of 28% (95%-CI [8%;43%]). The overall BLS performance of the intervention group was also significantly better than the control group with a mean difference of 15.44 points (95%-CI [21.049.83]), *p* < *.001*. In the CSA the undergraduates of the intervention group reported a significant higher learning gain.

**Conclusion:**

VR proved to be effective in enhancing process quality of BLS, therefore, the integration of VR into resuscitation trainings should be considered. Further research needs to explore which combination of instructional designs leads to deliberate practice and mastery learning of BLS.

## Introduction

Basic Life Support (BLS), performed by lay rescuers, is a significant determinant of patient outcome after sudden cardiac arrest, which is one of the major causes of death worldwide [1–4]. Next to recognizing cardiac arrest and alerting emergency medical services, the quality of cardiopulmonary resuscitation (CRP) is crucial [3, 5]. One key component of high quality CPR is that chest compressions are not interrupted- in order to maintain circulation of important organs. This key component is reflected in the “no-flow time”, which therefore, should be as minimal as possible [3, 6].

Although many educational efforts of the past years aimed to enhance lay rescuers BLS skills, the prognosis of out-of-hospital cardiac arrest (OHCA) remains low, with an estimated survival of about 10% worldwide [4, 7]. Therefore, the European Resuscitation Council (ERC) Guidelines 2021 highlight the role of BLS as one of the key strategies of OHCA survival [3, 5]. The implementation and expansion of educational strategies to enhance effectively BLS process quality, is therefore inevitable. So far, the widespread training method of BLS is the classic instructor-led mannequin training, conducted in small groups [8]. Further instructional designs and training approaches have been suggested, including Virtual Reality (VR) [5, 9–11]. VR is a computer generated simulation technique, which yields many benefits by providing a high level of immersion [12–15]. It provides a sheltered learning environment which enables trainees to experience virtual scenarios almost as in real life [16] and experience different clinical settings easily and flexibly [15]. As a result, autonomous learning takes place and improves contextualization of learning and hereby enhances learning outcomes. The application of VR in medical education has increased over the past years, [11, 14, 15] and the usability and acceptance of this teaching approach for BLS training has been confirmed [10, 11, 17, 18]. Especially in times of the Covid-19 pandemic, which lead to the closure of universities, disruption of face-to-face teaching and its replacement with virtual and web-based learning classes [19–21] the use of VR, particularly for procedural skills of BLS, is a promising solution. As it allows contact-teaching in very small groups under strict hygiene standards and COVID safe principles.

Although it is known, that VR improves learning outcomes in surgical skills [22], a recent ILCOR CoSTR (International Liaison Committee on Resuscitation Consensus on Science with Treatment Recommendations) systematic review identified a lack of evidence for the use of VR in resuscitation trainings (a.e. BLS), regarding skill performance and process quality [23]. Therefore, this randomized controlled trial, aimed to explore the effectiveness of a VR BLS training (intervention) on BLS learning outcomes, compared to web-based BLS training. It was hypothesised that the VR training was more effective than the web-based training in terms of no-flow time (primary outcome), and the overall quality of BLS (secondary outcome). Furthermore, the subjective learning gain of the participants for both training approaches was assessed (tertiary outcome).

## Methods

### Study design

This randomised, double-blinded controlled study was performed at the department of Anaesthesiology, University Medical Center Hamburg-Eppendorf, during the winter semester 2020/21. This study was reported in accordance to the CONSORT guidelines [24].

During their first semester of medical school, all 1^st^ year undergraduates are assigned to a mandatory BLS training, conducted by the department of anaesthesiology. Prior to the pandemic, this training included a theoretical part (seminar) and a hands-on mannequin-based practical training. During the pandemic, face-to-face teachings were interrupted or strictly modified (strict hygenic rules- a.e. not more than three persons in a room, limited number of students who were allowed to enter the university building per day). Therefore, the BLS training was replaced by a web-based training, which was broadcasted via *Cisco Webex™ Online Meetings, Milpitas, California, US*.

A maximum of twenty- one undergraduates were assigned for each training and a total of 19 trainings were conducted. Each web-based training was also composed of two parts: First, a 60-min seminar on BLS was held by one instructor, covering all the learning objectives as described by the European Resuscitation Council (ERC) Guidelines 2021 [25, 26]. The seminar was followed by an online demonstration (120 min) of BLS which was carried out by two instructors, using the Resusci Anne QCPR, Laedal, Stavanger, Norway. One of the instructors demonstrated the sequence of BLS, typical pitfalls and mistakes of chest compression, like wrong compression depth or frequency. During the demonstration, the undergraduates were talked through by the second instructor. The undergraduates were encouraged to practice the cardiopulmonary resuscitation on pillows at home.

The intervention group had an additional VR BLS training within a time span of three days after the web-based training. The VR training was composed of an introduction to the VR module (20 min) and a training unit (35 min). At the end of the VR training, the undergraduates performed a three-minutes structured clinical examination (SCE) on BLS, using the Resusci Anne QCPR (Laedal, Stavanger, Norway). The control group also took the SCE within the same time span after the web-based training. All SCEs were supervised by the same instructor, who made both groups familiar with the mannequin and its functions prior to each SCE, to reduce cognitive bias.

In summary, the main differnces between the training approaches were: Prior to the pandemic, the students participated in a face-to-face training, in which practical skills were directly rehearsed on mannequins. During the pandemic, this training was replaced by a mannequin-based online instruction, without the possibility of mannequin-based rehearsal. The VR training enabled the students to train the skills on the mannequin with direct feedback through the VR module.

### Participants

All first-year undergraduates (*N* = 360) were eligible for the study. Prerequisite for participation was the participation in the BLS web-based training prior to the intervention. Exclusion criteria were symptoms of illness (the undergraduates were not tested for COVID-19 on regular base at that time). An email with a detailed description of the study, the VR training and the possibility to apply, was sent to the 1^st^ year undergraduates two weeks prior to the semester.

A total of 120 VR slots were integrated in the teaching timetable of the undergraduates by the Dean´s office and the undergraduates had to apply for a VR training by sending an email to the the teaching coordinator of the department of anaesthesiology. The first 120 undergraduates who applied were enrolled within the study and received an VR appointment. Those who confirmed their appointment were randomised to the intervention- or control group (computer-generated random numbers). The undergraduates were blinded and and the allocation to the study groups was only documented by one instructor and was not disclosed to the undergraduates or to the assessors of the BLS checklist. The undergraduates were told to keep discrete about their training.

### Intervention

#### Virtual reality BLS-Training

**Fig. 1:**
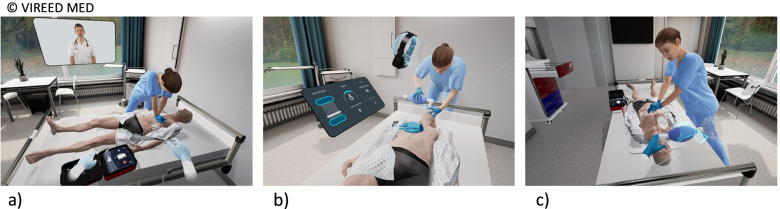
VR BLS training module. **a.** User as a passive observer in a patient room teacher. The BLS is provided by the clinical staff and the virtual educator describes and explains every step. **b.** User is an active BLS provider and carries out the BLS steps in a training modus. Direct feedback is provided for the chest compressions. **c.** The scenario is repeated and every step of BLS is carried out by the trainee without assistance

The individual VR training had a duration of 35 min and was supervised by the same instructor. The VR system and a pilot version of the software was developed by VIREED MED, Hamburg- Germany, a start-up company which was founded in 2017. With a research grand of the “Jung Foundation for Science and Research”, we were able to aquire the VR system as well as additional services of VIREED MED, which included tailoring the software to our requests and needs. The VR system is connected to a small CPR mannequin- and therefore, training of chest compressions is possible and direct feedback on the quality of chest compressions is visually provided (Fig. 1b). Bag-mask-ventilation and the use of an AED are virtually implemented in the system, but no actual haptic handling takes place.

The VR BLS training was composed of two sections: In the first section a correct BLS scenario was demonstrated and explained by a virtual teacher. After that, the participants had to manage and guide a BLS scenario together with a virtual collegue, who performed the chest compressions. In the second section, the undergraduates could practice chest-compressions on the mannequin and the virtual college provided the bag-mask-ventilation. Subsequently, they were confronted with a real-life emergency scenario in which they had to provide BLS without assistance. Figure 1 summarises the chronology and content of the VR BLS training.

### Outcomes

The primary outcome was the no-flow time, assessed in the three-minute structured clinical examination (SCE) by the Laerdal Skill Reporter Software (Laerdal, Stavenger, Norway).

The secondary outcome was the overall BLS performance, assessed by an adapted observational checklist which is used by the ERC and has been validated by Graham and Lewis in 2000 [27]. Each SCE was recorded, and afterwards independently analysed by two blinded assessors, who are experienced in BLS training and medical education.

The BLS checklist is composed of ten items (Table 1) and for each item penalty points can be given, according to pre-defined performance. Penalty points are awarded for incorrect performance of each BLS component, with reliance to the potential to compromise patient safety. The best possible BLS performance is combined with zero penalty points, the worst performance with 125 penalty points.

**Table 1:** Basic Life Support scoring system

Criterion	Value	Penalty points
1.Assessment	Right	0
	Wrong	5
2.Call for help	Right / done	0
	Wrong / not done	5
3.Open airway	Right	0
	Inadequate	10
	No attempt/ wrong	20
4.Assess breathing	Right assessment	0
	Inadequate assessment	10
	No assessment	20
5.Telephone for help	Yes	0
	No	20
6.CC (hand position)	Right	0
	Wrong	10
	Grossly wrong	20
7.CC (frequency)	100–120/ min	0
	-80–100/ min -120–140/ min	5
	- < 80/ min - > 140/min	15
8.CC (depth)	50–60 mm	0
	-42-59 mm -61-69 mm	10
	- < 42 mm - > 69 mm	20
9.CC (recoil)	- > 70%	0
	-70–33%	10
	- < 33%	20
10.AED	Used/asked for AED	0
	Not used/ not asked for AED	10

*Note: Adapted from Graham and Lewis, 2000. Abbreviations. CC* = *Chest compression. AED* = *Automatic external defibrillator*

The tertiary outcome was the learning gain which was assessed by a comparative self-assessment (CSA) [28], a validated self-assessment tool, which is composed of eleven questions (shown in Table 3.) that assess the learning gain of BLS. For each question a six-point Likert scale is provided (*1* = *mostly applies; 6* = *does not apply*).

The undergraduates filled out the CSA prior and after the intervention/control SCE.

The learning gain was computed with two methods. First according to the following formula which has been described by Raupach and colleges, in order to compute the learning gain in percentage [28].

CSA gain (%) = (CSA_pre_—CSA_post_) / (CSA_pre_—1) × 100.

In this method, participants who rated themselves with the highest possible score (1 = mostly apply) at the pre-test were so to speak “automatically” excluded from the analysis, because the term (CSA_pre_—1) leads to a division by zero, resulting in missing % learning gain values for these participants.

To compute differences in score points, a subtraction of the pre-intervention and post-intervention scores of all undergraduates was conducted:

CSA gain (points) = CSA_pre_—CSA_post_.

### Statistical analysis

Descriptive statistics were applied for the calculation of the mean values of the penalty points, given by the two assessors. The penalty points of each study group were compared applying an unpaired t-test. For the calculation of rater agreement (penalty points) the intraclass correlations (ICC) were computed, with a *two-way random effects model* (agreement definition). The ICC was interpretated according to Ciccetti: Values of ICC below 0.40 are interpreted as poor- values between 0.40 and 0.59 as a fair, between 0.59 and 0.75 as good- and values between 0.75 and 1 as an excellent correlation [29].

Sample size calculations using PASS 2008 version 08.0.6 [30] indicated that a sample size of 42 for each group achieves 81% power to detect superiority using a one-sided, two-sample t-test (assumptions: equivalence margin = 0, true ratio of the means = 0.9, α = 0.025, coefficients of variation of both groups = 0.17).

Histograms of data distributions of dependent variables (No Flow Time, CSA difference, % CSA gain) were visually examined by intervention group (and CSA item, if applicable). Their variances were computed by intervention group and assessed for homogeneity. Data values of No Flow Time (in seconds) were ln-transformed prior to further analyses because they were right-skewed. Which means the data were transformed to their natural logarithm (= ln). This transformation it is used to eliminate or reduce right-skewness in the data distribution, so the data fits better for general linear modeling.

A general linear model was fitted to the dependent variable (No-flow time)- with intervention group as a fixed effect. For the dependent variables CSA-difference and % CSA gain, a general linear mixed effects model was applied, considering participant as a random effect and CSA items with participants, as repeated measures.

CSA-difference were intervention group, CSA item and intervention group x CSA item, baseline-adjusting the analysis by CSA pre-rating used as a covariate. For the dependent variable % CSA gain, the same fixed effects were included in the model except CSA pre-rating. Model-estimated marginal means with 95% confidence intervals were computed and pairwise group comparisons were done. IBM SPSS version 27 was used for all statistical analyses employing its routine GENLINMIXED for the general linear (mixed) modelling work. A two tailed *p* < 0.05 was considered as statistically significant.

## Results

### Participants

**Fig. 2:**
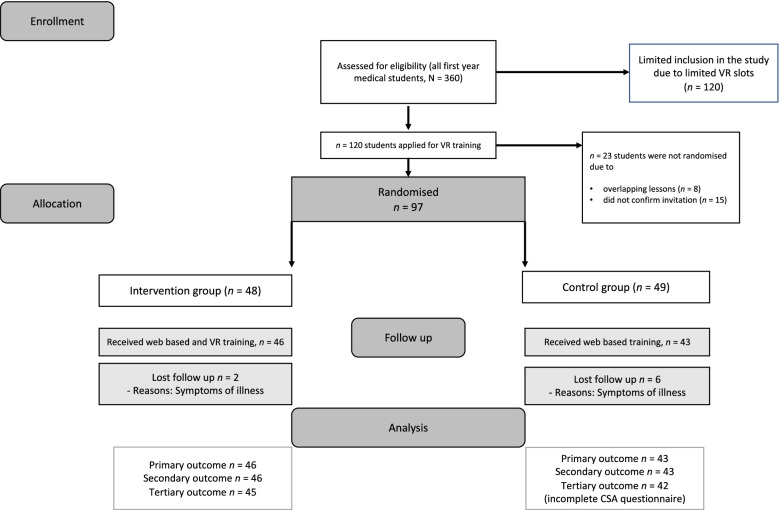
Participant flow of the study. No legend

The first *N* = 120 undergraduates who applied were enrolled in the study*, n* = 23 undergraduates had to be excluded due to overlapping lessons or due to lacking confirmation of the invitation. The remaining *n* = 97 undergraduates were randomised (*n* = 48 intervention-, *n* = 49 control group). Further eight undergraduates were excluded, because they had symptoms of illness (Fig. 2). Complete data from the Skill Reporter and videotapes were obtained from *n* = 89 SCEs (*n* = 46 intervention-, *n* = 43 control group). One CSA questionnaire (control group) had to be excluded due to missing data.

The demographic data of study particpants did not differ significantly, as shown in Table 2.

**Table 2:** Demographic data of study participants

	**Intervention group (*n* = 46)**	**Control group (*n* = 42)**	***p***
Age *M (range), yr*	20.50 (16–29)	20.98 (18–40)	.489
Gender, *n* (%)			
Female	33 (71.7)	29 (69)	.785
Male	13 (28.3)	13 (31	
Previous CPR experience, *n* (%)	8 (17.4)	7 (16.7)	.929
Previous VR experience, *n* (%)	2 (4.3)	5 (11.9)	.195

*Abbreviations. Yr* *Year. CPR* *Cardiopulmonary resuscitation. VR* *Virtual reality*

### Primary outcome: No-flow time

The overall no-flow time, assessed with the Laerdal Skills Reporter (Laedal, Stavanger, Norway) during the SCEs, was *M* = 8.65 s (*SD* = 10.761). The no-flow time of the intervention group was significantly lower (*M* = 6.46 s, *SD* = 3.49) than in the control group (*M* = 11.05 s, *SD* = 14.89). Back-transformed marginal means of no-flow time estimated by a general linear model were 5.80 (95%-CI [4.91;6.86]) for the intervention group and 8.04 (95%-CI [6.75;9.58]) for the control group (*p* = 0.009), indicating a difference between the two groups of about 28% (95%-CI [8%;43%]).

### Secondary Outcome: Overall BLS performance

The intervention group received significantly lower penalty points on the BLS checklist (*M* = 13.75, *SD* = 9.66) than the control group (*M* = 29.19, *SD* = 16.31), with a mean difference of 15.44 points (95%-CI [-21.16; -9.72]), *t (67.3)* = *-5.39, p* < *0.001.*

The interrater reliability showed a good agreement between the ratings of the two independent raters: *ICC* 0.76 (95%-CI [0.63; 0.85]).

### Tertiary outcome: CSA- subjective learning gain

**Fig. 3:**
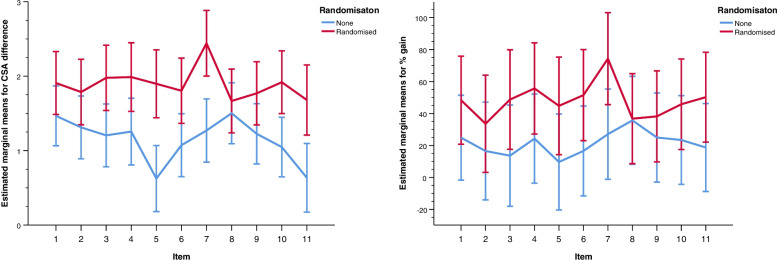
depicts the percentage of learning gain as well as the gain expressed in points. Learning gain of undergraduates assessed with the CSA. *Note*: The left graph panel depicts the estimated marginal means with 95% confidence intervals of CSA gain points (y-axis) for all undergraduates. The right graph panel depicts model estimated marginal means with 95% confidence intervals of % CSA gains (y-axis) calculated by the Göttingen method (Raupach et al.). The numbers of the x-achsis represent individual CSA items

The undergraduates who participated in the VR training reported significantly higher learning gains than the control group for all items of the CSA, except for item 8 (“I feel competent about the correct sequence of treatment of BLS”) (Table 3, Fig. 3). The highest learning gain (over 50%), was reported for items 4, 6, 7 and 11. The greatest difference was reported for item 5 (“I feel confident to provide mask ventilation”). For item 8 (“I feel confident about the correct sequence of treatment of BLS”) the difference between the groups was at lowest.

**Table 3:** Subjective learning gain reported by the undergraduates

Items		Intervention *n* = 46 Control *n* = 42	Mean learning gain in points	Mean learning gain in percent	*p* (difference in points)	*p* (difference in percent)
1*	I feel confident to provide BLS	Intervention	2.99	48.32	.026	.067
		Control	2.52	24.89		
2*	I feel confident to detect an irregular breathing	Intervention	2.87	33.60	.028	.298
		Control	2.4	16.53		
3*	I feel confident to detect a cardiac arrest	Intervention	3.06	48.73	< .001	.043
		Control	2.29	13.64		
4*	I feel confident to clear the patient´s airway	Intervention	3.07	55.68	.003	.027
		Control	2.34	24.32		
5*	I feel confident to provide mask ventilation	Intervention	2.98	44.77	< .001	.032
		Control	1.71	9.67		
6*	I feel confident to perform high quality chest compressions	Intervention	2.89	51.50	.001	.015
		Control	2.16	16.58		
7*	I feel confident with the use of the AED	Intervention	3.53	74.33	< .001	.001
		Control	2.36	27.07		
8	I feel confident about the correct sequence of treatments of BLS	Intervention	2.76	36.77	.421	.938
		Control	2.59	35.71		
9*	A person lies motionless on the street. I feel confident being able to provide BLS	Intervention	2.86	38.20	.007	.345
		Control	2.31	24.98		
10*	The patient lies motionless in his bed. I feel confident being able to provide BLS	Intervention	3.00	45.80	< .001	.108
		Control	2.13	23.43		
11*	I feel able to lead BLS in a team	Intervention	2.76	50.21	< .001	.022
		Control	1.72	18.73		

* marks the items for which the students reported significant different learning gains and are pairwise comparisons. Abbreviations. BLS = *Basic life support. AED* = *Automatic external defibrillator*

## Discussion

In this randomised controlled trial, a VR BLS training significantly improved the BLS performance of 1^st^ year medical undergraduates, compared to a web-based CPR training, with respect to no-flow time (primary outcome) and overall performance (secondary outcome). Furthermore, the VR training led to a better self-perceived learning gain of BLS related skills, assessed with the comparative self-assessment (tertiary outcome).

The goal of CPR is the maintenance of organ perfusion and accordingly the reduction of ischemic injury. Therefore, next to the overall correctness of chest compression (frequency, depth, recoil), the no-flow time can be considered as a sensitive indicator of BLS quality [3], as reduced no-flow times are associated with increased survival after OHCA [31]. So far, there are no evidence-based recommendations for deliberate practice and mastery learning of BLS [32] and only few published studies explored the effectiveness of VR CPR training on procedural skills and brought inconclusive evidence [9–11]. This gap in knowledge was also confirmed by a recent ILCOR CoSTR review [23]. The present study contributes to the current evidence on VR based CPR training and supports its effectiveness on process quality of BLS. Therefore, especially during the Covid-19 pandemic, we recommend, to embed VR in CPR trainings, as a feasible way of teaching BLS when face to face mannequin group teaching is not possible. The results from the CSA also confirm the effectiveness of VR in BLS trainings. Although based on self-perception, the learning gain can be considered as an indirect parameter for actual skill enhancement, as good correlations between subjective learning gain (CSA) and objective learning gain, measured with summative assessment, have been described [33]. A positive side-effect of self-perceived skill improvement is the increased willingness to perform bystander CPR [34], which is a significant determinant to improve survival after OHCA and correlates with a threefold increase of surivival and enhanced neurological outcome [35–37]. According to the European Registry of Cardiac Arrest (EuReCa), the bystander CPR rate is estimated at 58% in Europe [38] and needs to be improved. Thus, the use of VR in CPR trainings could contribute to trainees´ confidence and therefore increase their willingness to perform bystander CPR [34].

Interestingly, the VR group reported the highest learning gain for item seven (“I feel competent with the use of the AED”) and the learning gain was even three-fold higher than in the control group. Neither of the groups had used or connected the Automated Exernal Defibrillator (AED) in a real-life setting before. Although we put maximum effort to develop an ideal didactic concept for the web based training, stimulating the interactive learning phases [39], by applying peyton´s approach (particularly step three: trainee talks the trainer through the procedure) [40] for the use of an AED, the VR stimulated AED use still lead to a higher learning gain, although it was only virtually performed. This highlights the supportive influence of immersion on acceleration of the learning cycle [15]. Nevertheless, the value of classical teaching approaches should not be underestimated, as for theoretical aspects, like the sequence of BLS (item 8 of the CSA, Table 3), the learning gain of both groups was comparable.

Some limitations of this study merit consideration, based on constructive alignment, the results are not surprising, as the learning of technical skills, like chest compression, should be taught by practical training and demonstration [41]. Therefore, one might argue that the study design itself presupposed the results and questions the generalisability of the positive training effects of VR BLS training on BLS quality. Nevertheless, during the COVID-19 pandemic and the forced disruption of mannequin-based trainings, no other alternative to the web-based training than the VR training was given at our university to convey BLS. This points out further liminations: As the VR training required access to specific equipment, the number of students who had the chance to participate in the training was limited. An alternative without any restrictions regarding participants´ number would have been remote education, which involves educators and students who are not accustomed to education that takes place online. Neverthelss, to our best knowledge no general accessible or affordable remote educational program is yet developed to connect practical skills with direct feedback at a high level of immersion for the training of BLS. Therefore, remote training possibilities- combining practical skills and theory- for the purpose of training BLS should be developed and evaluated, as independent from the pandemic, remote training possibilities would allow a less restricted and a far more flexible learning. The greatest obstacle for remote education of BLS arises from its costs- therefore, remote training possibilities need to become more easily accessible and more favourable.

Detached from pandemic circumstanes, the results are still of value, as the no-flow time was defined as the primary endpoint. The no-flow time is not an actual haptic skill but more a procedural skill which is a translation of situational awareness into behaviour [42], and not necessarily trained in classical CPR trainings (mannequin-/simulation-based) [43]. Therefore, the objection can be ruled out, that the online teaching was not sufficient to create a sense of awareness for the crucial impact of the no-flow time. Furthermore, one might argue that the VR group profited from the additional training, as they had also participated in the web-based training. This is a further limitation of the study design: The control group could have yielded similar results with a repeated use of the web-based training through reinforcement. This limitation might compromise the conclusion that the VR training was more effective than the web-based training but the conclusion that the VR training enhanced effectively BLS skills is applicable. Future trainings must adapt these findings, and we suggest, that in times or places where no hands-on-training is possible, the best way to train BLS will be a combination of a web-based seminar on the theory of cardiac arrest, followed by a VR-BLS-training.

The second outcome was assessed with a checklist which rated the overall BLS performance. Although this checklist is used by the ERC and has been validated [27], it is known that several effects can influence and bias assessor´s SCE ratings [44]. To minimise bias of the ratings, we chose assessors with similar characteristics [45]. Furthermore, the assessors rated every SCE video independently and the interrater reliability of the ratings were good, assuming that the results of the overall BLS performance are valid.

## Conclusion

VR based CPR training enhances process quality of BLS compared to pandemic determined web-based teaching, and leads to an improved subjective learning gain, which in turn indicates increased willingness to perform bystander CPR. As 1^st^ year medical students can be considered as lay persons, our results on the effectiveness of VR training is transferable to all lay trainees. Therefore, we recommend the application of VR during the pandemic and moreover, detached from the pandemic, the broad use of VR in CPR training should be considered to complement classical trainings, as VR seems to be a promising approach to deliberate practice and mastery learning. Further research should explore the effectiveness of VR CPR training in comparison with non-pandemic mannequin-/simulation-based trainings.

## Data Availability

The datasets used and/or analysed during the current study are available from the corresponding author on reasonable request.

## Abbreviations

VR: Virtual Reality
BLS: Basic Life Support
CRP: Cardiopulmonary resuscitation
OHCA: Out-of-hospital cardiac arrest
ERC: European Resuscitation Council
ILCOR CoSTR: Internation Liaison Committee Consensus on Science with Treatment Recommendations
SCE: Structured clinical examination
CSA: Comparative self-assessment
ICC: Intraclass correlations
AED: Automatic external defibrillator

## References

[1] Priori SG, Blomström-Lundqvist C, Mazzanti A, Blom N, Borggrefe M, Camm J, et al. 2015 ESC Guidelines for the management of patients with ventricular arrhythmias and the prevention of sudden cardiac Death. The Task Force for the Management of Patients with Ventricular Arrhythmias and the Prevention of Sudden Cardiac Death of the European Society of Cardiology. Giornale italiano di cardiologia (2006). 2016;17(2):108–70. 10.1714/2174.23496 27029760

[2] Perkins GD, Graesner JT, Semeraro F, Olasveengen T, Soar J, Lott C (2021). European resuscitation council guidelines 2021: executive summary. Resuscitation..

[3] Olasveengen TM, Semeraro F, Ristagno G, Castren M, Handley A, Kuzovlev A, et al. European resuscitation council guidelines 2021: basic life support. Resuscitation. 2021;161:98–114. ISO 690. 10.1016/j.resuscitation.2021.02.009 33773835

[4] Gräsner JT, Herlitz J, Tjelmeland IB, Wnent J, Masterson S, Lilja G (2021). European Resuscitation Council Guidelines 2021: epidemiology of cardiac arrest in Europe. Resuscitation..

[5] Greif R, Lockey A, Breckwoldt J, Carmona F, Conaghan P, Kuzovlev A (2021). European resuscitation council guidelines 2021: education for resuscitation. Resuscitation.

[6] Soar J, Nolan JP, Böttiger BW, Perkins GD, Lott C, Carli P (2015). European resuscitation council guidelines for resuscitation 2015: section 3. Adult advanced life support. Resuscitation.

[7] Benjamin EJ, Muntner P, Alonso A, Bittencourt MS, Callaway CW, Carson AP (2019). Heart disease and stroke statistics—2019 update: a report from the American Heart Association. Circulation.

[8] Perkins GD (2007). Simulation in resuscitation training. Resuscitation.

[9] Issleib M, Kromer A, Pinnschmidt HO, Süss-Havemann C, Kubitz JC (2021). Virtual reality as a teaching method for resuscitation training in undergraduate first year medical students: a randomized controlled trial. Scandinavian journal of trauma, resuscitation and emergency medicine.

[10] Aksoy E (2019). Comparing the effects on learning outcomes of tablet-based and virtual reality–based serious gaming modules for basic life support training: randomized trial. JMIR serious games.

[11] Semeraro F, Ristagno G, Giulini G, Gnudi T, Kayal JS, Monesi A (2019). Virtual reality cardiopulmonary resuscitation (CPR): Comparison with a standard CPR training mannequin. Resuscitation.

[12] Gaddis T (1997). Using Virtual Reality To Bring Your Instruction to Life.

[13] Mantovani F (2001). 12 VR learning: potential and challenges for the use of 3D. Towards Cyberpsychol..

[14] Hussein M, Nätterdal C. The benefits of virtual reality in education-a comparision study. 2015.

[15] Pottle J (2019). Virtual reality and the transformation of medical education. Future healthcare journal.

[16] McGrath JL, Taekman JM, Dev P, Danforth DR, Mohan D, Kman N (2018). Using virtual reality simulation environments to assess competence for emergency medicine learners. Acad Emerg Med.

[17] Ingrassia PL, Mormando G, Giudici E, Strada F, Carfagna F, Lamberti F (2020). Augmented reality learning environment for basic life support and defibrillation training: usability study. J Med Internet Res.

[18] Bench S, Winter C, Francis G (2019). Use of a virtual reality device for basic life support training: Prototype testing and an exploration of users' views and experience. Simulation in Healthcare.

[19] Rose S (2020). Medical student education in the time of COVID-19. JAMA.

[20] Ahmed H, Allaf M, Elghazaly H (2020). COVID-19 and medical education. Lancet Infect Dis.

[21] Almarzooq ZI, Lopes M, Kochar A (2020). Virtual learning during the COVID-19 pandemic: a disruptive technology in graduate medical education. J Am Coll Cardiol.

[22] Barsom EZ, Graafland M, Schijven MP (2016). Systematic review on the effectiveness of augmented reality applications in medical training. Surg Endosc.

[23] Greif R, Bhanji F, Bigham BL, Bray J, Breckwoldt J, Cheng A, et al. Education, implementation, and teams: 2020 international consensus on cardiopulmonary resuscitation and emergency cardiovascular care science with treatment recommendations. Circulation. 2020;142(16_suppl_1):S222–83. 10.1161/CIR.0000000000000896 33084395

[24] Bennett JA (2005). The consolidated standards of reporting trials (CONSORT): Guidelines for reporting randomized trials. Nurs Res.

[25] Olasveengen TM, Mancini ME, Perkins GD, Avis S, Brooks S, Castrén M, et al. Adult basic life support: 2020 international consensus on cardiopulmonary resuscitation and emergency cardiovascular care science with treatment recommendations. Circulation. 2020;142(16_suppl_1):S41–91. 10.1161/CIR.0000000000000892 33084391

[26] Perkins GD, Handley AJ, Koster RW, Castrén M, Smyth MA, Olasveengen T, et al. European resuscitation council guidelines for resuscitation 2015: section 2. Adult basic life support and automated external defibrillation. Resuscitation. 2015;95:81–99. 10.1016/j.resuscitation.2015.07.015 26477420

[27] Graham CA, Lewis NF (2000). A scoring system for the assessment of basic life support ability. Resuscitation.

[28] Raupach T, Münscher C, Beissbarth T, Burckhardt G, Pukrop T (2011). Towards outcome-based programme evaluation: using student comparative self-assessments to determine teaching effectiveness. Med Teach.

[29] Cicchetti DV (1994). Guidelines, criteria, and rules of thumb for evaluating normed and standardized assessment instruments in psychology. Psychol Assess.

[30] Hintze JL (2008). Quick start manual.

[31] Bobrow BJ, Clark LL, Ewy GA, Chikani V, Sanders AB, Berg RA (2008). Minimally interrupted cardiac resuscitation by emergency medical services for out-of-hospital cardiac arrest. JAMA.

[32] Greif R, Bhanji F, Bigham BL, Bray J, Breckwoldt J, Cheng A (2020). Education, Implementation, and Teams: 2020 International Consensus on Cardiopulmonary Resuscitation and Emergency Cardiovascular Care Science With Treatment Recommendations. Resuscitation.

[33] Schiekirka S, Reinhardt D, Beibarth T, Anders S, Pukrop T, Raupach T (2013). Estimating learning outcomes from pre-and posttest student self-assessments: a longitudinal study. Acad Med.

[34] Cho GC, Sohn YD, Kang KH, Lee WW, Lim KS, Kim W (2010). The effect of basic life support education on laypersons’ willingness in performing bystander hands only cardiopulmonary resuscitation. Resuscitation.

[35] Hasselqvist-Ax I, Riva G, Herlitz J, Rosenqvist M, Hollenberg J, Nordberg P (2015). Early cardiopulmonary resuscitation in out-of-hospital cardiac arrest. N Engl J Med.

[36] Christensen D, Rajan S, Kragholm K, Søndergaard K, Hansen O, Gerds T (2019). Bystander cardiopulmonary resuscitation and survival in patients with out-of-hospital cardiac arrest of non-cardiac origin. Resuscitation.

[37] Sasson C, Rogers MA, Dahl J, Kellermann AL. Predictors of survival from out-of-hospital cardiac arrest: a systematic review and meta-analysis. Circulation. 2010;3(1):63–81. ISO 690. 10.1161/CIRCOUTCOMES.109.889576 20123673

[38] Gräsner J-T, Wnent J, Herlitz J, Perkins GD, Lefering R, Tjelmeland I (2020). Survival after out-of-hospital cardiac arrest in Europe-Results of the EuReCa TWO study. Resuscitation.

[39] Chi MT, Wylie R (2014). The ICAP framework: Linking cognitive engagement to active learning outcomes. Educational Psychologist.

[40] Peyton JWR. Teaching in theatre. Teaching and learning in medical practice. Manticore. 1998:171–80.

[41] Biggs J. Constructive alignment. In Background notes to support a seminar given by Professor John Biggs. jbiggs@ bigpond. com ASSESSING LANGUAGE OR CONTENT. 2001.

[42] Flin R, Patey R, Glavin R, Maran N (2010). Anaesthetists' non-technical skills. Br J Anaesth.

[43] Zausig Y, Grube C, Boeker-Blum T, Busch C, Bayer Y, Sinner B (2009). Inefficacy of simulator-based training on anaesthesiologists' non-technical skills. Acta Anaesthesiol Scand.

[44] Mihevc M, Masnik K, Petreski T, Pulko N, Bevc S. FACTORS INFLUENCING ASSESSOR ‘S CHECKLIST AND GLOBAL SCORES AT OSCE.

[45] Zimmermann P, Kadmon M. Standardized Examinees: Development of a new tool to evaluate factors influencing OSCE scores and to train examiners. GMS J Med Educ. 2020;37(4). 10.3205/zma001333 PMC7346289 32685668

